# Construction of Dual-Biofunctionalized Chitosan/Collagen Scaffolds for Simultaneous Neovascularization and Nerve Regeneration

**DOI:** 10.34133/2020/2603048

**Published:** 2020-08-10

**Authors:** Guicai Li, Qi Han, Panjian Lu, Liling Zhang, Yuezhou Zhang, Shiyu Chen, Ping Zhang, Luzhong Zhang, Wenguo Cui, Hongkui Wang, Hongbo Zhang

**Affiliations:** ^1^Key Laboratory of Neuroregeneration of Jiangsu and Ministry of Education, Nantong University, 226001 Nantong, China; ^2^Co-Innovation Center of Neuroregeneration, Nantong University, 226001 Nantong, China; ^3^Frontiers Science Center for Flexible Electronics, Xi'an Institute of Flexible Electronics (IFE) & Xi'an Institute of Biomedical Materials and Engineering (IBME), Northwestern Polytechnical University, 127 West Youyi Road, Xi'an 710072, China; ^4^Shanghai Institute of Traumatology and Orthopaedics, Shanghai Key Laboratory for Prevention and Treatment of Bone and Joint Diseases, Ruijin Hospital, Shanghai Jiao Tong University School of Medicine, 197 Ruijin 2nd Road, Shanghai 200025, China; ^5^Pharmaceutical Sciences Laboratory and Turku Bioscience Centre, Åbo Akademi University, 20520 Turku, Finland

## Abstract

Biofunctionalization of artificial nerve implants by incorporation of specific bioactive factors has greatly enhanced the success of grafting procedures for peripheral nerve regeneration. However, most studies on novel biofunctionalized implants have emphasized the promotion of neuronal and axonal repair over vascularization, a process critical for long-term functional restoration. We constructed a dual-biofunctionalized chitosan/collagen composite scaffold with Ile-Lys-Val-Ala-Val (IKVAV) and vascular endothelial growth factor (VEGF) by combining solution blending, *in situ* lyophilization, and surface biomodification. Immobilization of VEGF and IKVAV on the scaffolds was confirmed both qualitatively by staining and quantitatively by ELISA. Various single- and dual-biofunctionalized scaffolds were compared for the promotion of endothelial cell (EC) and Schwann cell (SC) proliferation as well as the induction of angiogenic and neuroregeneration-associated genes by these cells in culture. The efficacy of these scaffolds for vascularization was evaluated by implantation in chicken embryos, while functional repair capacity *in vivo* was assessed in rats subjected to a 10 mm sciatic nerve injury. Dual-biofunctionalized scaffolds supported robust EC and SC proliferation and upregulated the expression levels of multiple genes and proteins related to neuroregeneration and vascularization. Dual-biofunctionalized scaffolds demonstrated superior vascularization induction in embryos and greater promotion of vascularization, myelination, and functional recovery in rats. These findings support the clinical potential of VEGF/IKVAV dual-biofunctionalized chitosan/collagen composite scaffolds for facilitating peripheral nerve regeneration, making it an attractive candidate for repairing critical nerve defect. The study may provide a critical experimental and theoretical basis for the development and design of new artificial nerve implants with excellent biological performance.

## 1. Introduction

Accidental peripheral nerve trauma and degenerative disease processes can negatively impact life and health by impairing movement and other bodily functions and by inducing chronic pain [1]. Currently, autologous grafts are the “gold standard” for treating peripheral nerve damage, but broad application and clinical success may be limited by problems such as size mismatch between the graft and repair site, a lack of sufficient donor tissue, and permanent functional loss at the donor site. As an alternative, artificial nerve implants made from various natural or synthetic polymers have been developed and successfully applied to nerve regeneration [2, 3]. However, the clinical utility of these artificial implants for defects larger than 3 cm is still poor [4, 5]. One likely reason for regeneration failure is insufficient vascular network formation around or inside the implant [6], resulting in inadequate nutrient supply and oxygen-carbon dioxide exchange to support nerve growth. In addition, implants may not provide the appropriate microenvironment for nerve regeneration [7]. Therefore, implants should be endowed with both angiogenic and neuroregenerative capacity for optimal clinical efficacy.

Numerous implant constructs, including composite biomaterial scaffolds with angiogenesis induction capacity, scaffolds preseeded with induced stem cells, scaffolds with 3D-printed vascular networks, and scaffolds with immobilized angiogenic biofactors or genes, have been developed for various applications in tissue engineering and regenerative medicine [8–11]. While substantial progress has been achieved in scaffold vascularization [12], there are still inadequacies for clinical application. For example, contamination may occur when preseeding cells *in vitro*, and cell death following implantation may induce an inflammatory response. Biomaterials have been developed for 3D-printed vascular-like structures with excellent flexibility and plasticity, but model design and printing are complex and time-consuming. Moreover, the permeability of vascular networks formed via 3D printing is low compared to that of natural vascular networks, which may limit nutrient supply and gas exchange. Promising alternatives currently garnering great interest among researchers and clinicians are scaffolds with immobilized angiogenesis-promoting biofactors that eliminate the need for cell seeding. In fact, several angiogenesis-related biofactors have been identified for constructing vascularized tissue-engineered scaffolds [13–15]. Vascular endothelial growth factor (VEGF) is the most widely studied biofactor for enhancing the angiogenic potential of scaffolds. VEGF potently stimulates angiogenesis by inducing EC motility via binding to neuropilin 1 [16]. Indeed, acellular spinal cord scaffolds (ASCSs) with VEGF encapsulated poly(lactic-co-glycolic acid) (PLGA) nanoparticles (NPs) conjugated by genipin (GP) accelerated vascular reconstruction in rats with spinal cord injury [17], while collagen scaffolds with VEGF immobilized via its collagen-binding domain (CBD) promoted peripheral nerve regeneration and neovascularization in the model [18]. Therefore, incorporating VEGF has shown promise for improving the angiogenic capacity of nerve implants, but few studies on vascularized nerve tissue constructs have been conducted.

In addition to factors promoting vascularization, effective scaffolds require physical, chemical, or biological cues to guide the regeneration process, such as binding sites or microenvironments permissive to nerve cell attachment and migration [19–21]. Numerous biomolecules (such as growth factors and neurotrophins) with bioactivity for promoting cell attachment, proliferation, and differentiation have been tested for nerve regeneration potential [22, 23]. The extracellular matrix protein laminin has a well-described positive influence on cell growth and so is often used as a coating protein for scaffolds during cell seeding. The main binding site for neural cell laminin receptors is the cationic sequence IKVAV of the laminin *α*1 chain (termed the minimum recognition sequence). In turn, ligand-bound (activated) laminin receptors promote cell-substrate attachment, cell migration, and neurite outgrowth [24, 25]. Nanoscaffolds containing IKVAV significantly improved functional recovery after traumatic brain injury (TBI) [26], and nanofibers containing IKVAV blended with PLGA effectively promoted nerve and SC attachment and migration. Moreover, a PLGA/IKVAV-modified conduit was shown to promote axonal regeneration and functional recovery following creation of a 15 cm sciatic nerve gap [27]. Thus, IKVAV shows promise for functionalizing various biomaterial scaffolds to promote nerve regeneration at severe injury sites.

As VEGF plays a critical role in the formation of neovascular networks while IKVAV is a promising candidate for promoting Schwann cell growth and nerve regeneration, we speculated that the combination of immobilized VEGF and IKVAV may promote both vascularization and neuroregeneration for the repair of large peripheral nerve deficits. However, no report has examined the potential synergistic effects of VEGF and IKVAV coimmobilized on biomaterial scaffolds. Two of the main reasons are the instability and unsustainable release of growth factors such as VEGF due to weak binding with most scaffold materials. Thus, we require a technique for stable immobilization of VEGF and IKVAV, preferably in one step. Heparin is a highly sulfated anionic polysaccharide widely used as a clinical anticoagulant [28]. Heparin not only binds specifically to many growth factors with heparin-binding domains [29, 30] but also binds to a variety of proteins without specific binding domains via electrostatic interactions [31]. Several growth factors, including VEGF and basic fibroblast growth factor (bFGF), were reported to be effectively delivered to sites of injury via heparinized poly(D, L-lactic-co-glycolic acid) (PLGA) microspheres and nanoparticles [32]. In addition, our previous study demonstrated that chitosan scaffolds conjugated to heparin via electrostatic attraction can successfully load nerve growth factor (NGF) [7]. Further, the bioactivity of various growth factors may be better maintained by electrostatic binding compared to chemical conjugation. Considering the specific binding to VEGF and electrostatic attraction to IKVAV, heparin may be an ideal candidate for simultaneously immobilizing VEGF and IKVAV on biomaterial scaffolds.

Therefore, in the present study, chitosan/collagen composite scaffolds biofunctionalized with VEGF and IKVAV were fabricated by combined solution blending, in situ lyophilization, and surface biomodification. Chitosan and collagen were used in this study due to their excellent biocompatibility and demonstrated performance for various applications in tissue engineering and regenerative medicine. We first examined the physiochemical properties of dual-biofunctionalized chitosan/collagen scaffolds with various component ratios by chemical staining, electron microscopy, and UV spectral analysis. The immobilization of VEGF and IKVAV on the scaffolds was confirmed by immunostaining and ELISA. The effects of the prepared scaffolds on the attachment, spread, morphology, and gene expression profiles of ECs and SCs were evaluated in culture. Finally, the reparative efficacy of dual-biofunctionalized chitosan/collagen scaffolds was examined on 10 mm sciatic nerve injury via both morphological characterization and functional recovery tests. To the best of our knowledge, this is the first study demonstrating the synergetic effects of VEGF/IKVAV dual-biofunctionalized biomaterial scaffolds on both neuroregeneration and vascularization.

## 2. Results

### 2.1. Dual-Biofunctionalized Scaffold Preparation

Figure S2A presents the UV spectra before and after blending of chitosan (CS) and collagen (CO). All solutions displayed absorbance peaks around 315 nm, with CO exhibiting the weakest absorbance, CS exhibiting the strongest absorbance, and chitosan/collagen (CC) absorbance intermediate between CO and CS, suggesting successful complex formation. We further characterized CS-CO complex formation and heparin modification using toluidine blue (TBO) staining (Figure S2B-D). Notably, CS scaffolds showed no color variation after TBO staining, while CCH scaffolds were stained with the deepest blue. The scaffold color increased in the order: CS<CC (chitosan/collagen scaffolds)<CO<CCH (heparinized chitosan/collagen scaffolds). After the addition of ethanol/NaOH solution, TBO was diluted and showed purple color. The same trend of color variation was observed using (purple) acidified TBO. These results indicate that CC scaffolds can be successfully fabricated and further modified with heparin.

### 2.2. Physiochemical Characterization of Dual-Biofunctionalized Scaffolds

**Figure 1:**
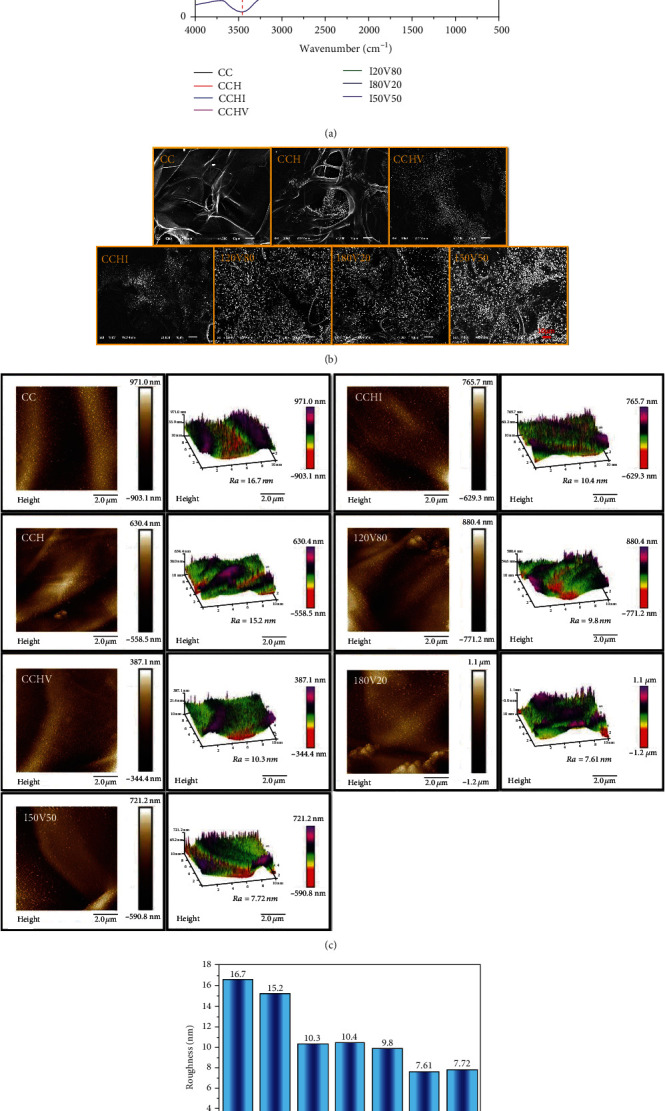
Physiochemical characterization of the scaffolds: (a) component variation by Fourier transform infrared spectrometry (FTIR), (b) morphology observation by scanning electron microscopy (SEM) (scale bar = 10 *μ*m), and (c, d) roughness detection by atomic force microscopy (AFM) scanning.

Figure 1(a) shows the structural and component variation of the scaffolds as a function of heparin modification and VEGF/IKVAV immobilization. The CC scaffolds exhibited obvious absorbance peaks at 3510 cm^−1^ corresponding to stretching vibration of the –OH group, 2912 cm^−1^ and 2850 cm^−1^ corresponding to stretching vibration of the –NH and -CH groups, 1635 cm^−1^ and 1650 cm^−1^ corresponding to shear vibration of the amide band, and 1538 cm^−1^ corresponding to stretching vibration of chitosan-NH belonging to chitosan. After heparin modification, the peaks at 3510 cm^−1^ displayed an obvious red shift to 3455 cm^−1^, while peaks at 1538 cm^−1^ decreased substantially in amplitude. After further VEGF/IKVAV immobilization, all the peaks at 2912 cm^−1^, 2850 cm^−1^, and 1538 cm^−1^ disappeared. But the intensity of adsorption peaks at 1635 cm^−1^ was markedly enhanced compared to that of CC and CCH scaffolds due to the overlap of stretching vibration of the amide band belonging to collagen, IKVAV, and VEGF. Notably, no obvious difference of Fourier transform infrared spectra (FTIR) was observed for all VEGF/IKVAV-immobilized CCH scaffolds compared to the bare scaffolds due to the overlap of the same functional groups. The morphology observation was further used to detect the variation of scaffolds during the dual-biofunctional process. Scanning electron microscopy (SEM) was then employed to examine morphological changes in scaffolds during the dual-biofunctionalization process. Bare CC scaffolds had smooth surfaces with fiber- or strip-like structures (Figure 1(b)) while the surfaces of CCH scaffolds exhibited visible absorbed particles. After immobilization of VEGF/IKVAV, a much denser aggregation of surface particles was observed. In addition, VEGF/IKVAV-immobilized scaffolds with three different VEGF : IKVAV ratios exhibited similar surface morphologies. Atomic force microscopy (AFM) in Figure 1(c) indicated that bare CC scaffolds had the greatest surface roughness (16.7 nm) and that heparin modification did not obviously affect roughness, while the roughness decreased significantly after VEGF/IKVAV immobilization. The rank order of roughness was CC>CCH>CCHI (CCH scaffolds grafted with IKVAV)≈CCHV (CCH scaffolds grafted with VEGF)>I20V80>I80V20>I50V50, where the digits indicate the IKVAV : VEGF ratio. Collectively, FTIR, SEM, and AFM indicated that dual biofunctionalization influences the surface composition and morphology of CCH scaffolds.

### 2.3. Confirmation of VEGF/IKVAV Immobilization and Release

The immobilization and release kinetics of VEGF and IKVAV were characterized quantitatively by the enzyme linked immunosorbent assay (ELISA) and qualitatively using immunofluorescence staining. Figures S3A and S3B show the amounts immobilized while Figures S3D and S3E show the distributions of VEGF and IKVAV in the scaffolds. As expected, ELISA demonstrated that the VEGF-loaded scaffolds CCHV and I20V80 had significantly more immobilized VEGF than CCH, CCHV, I80V20, and I50V50 scaffolds (*P* < 0.05), while the IKVAV-loaded scaffolds CCHI and I80V20 had significantly more immobilized IKVAV than CCH, I20V80, I50V50, and CCHV scaffolds (*P* < 0.05). Immunofluorescence staining with subsequent intensity measurements further verified these results. Following immersion in PBS, scaffolds released both VEGF and IKVAV (Figures S3C and S3F), reaching equilibria after about 1 h with no further increase in release during longer immersion. Scaffolds without VEGF or IKVAV immobilization showed significantly lower amounts of release (*P* < 0.05), while no differences were observed among the scaffolds with different VEGF or IKVAV ratios. Thus, both VEGF and IKVAV can be immobilized in scaffolds to confer prolonged stable release.

### 2.4. Dual-Biofunctionalized Scaffolds Enhance Schwann Cell and Endothelial Cell Proliferation and Viability

**Figure 2:**
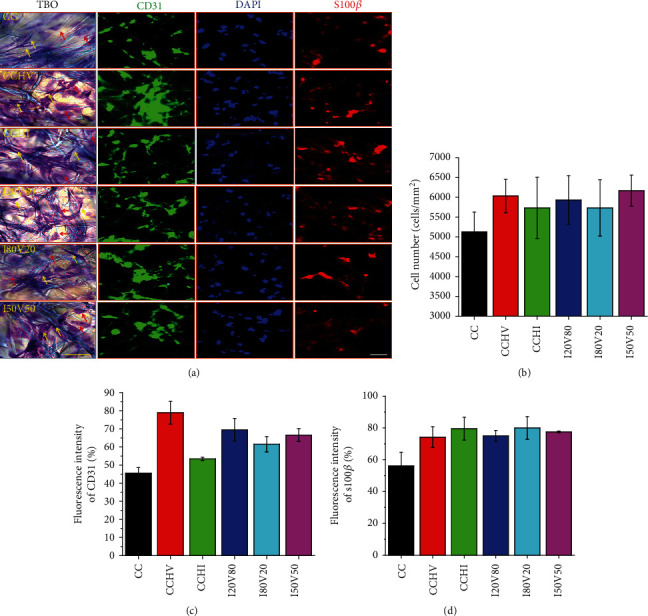
Coculture of endothelial cells (ECs) and Schwann cells (SCs) in various scaffolds: (a) toluidine blue (TBO) staining and immunofluorescence staining using platelet endothelial cell adhesion molecule-1 (CD31), 4′,6-diamidino-2-phenylindole (DAPI), and s100*β* after coculture for 3 days; (b) total number of ECs and SCs in all scaffolds; (c, d) fluorescence intensity of CD31 and s100*β* corresponding to ECs and SCs, respectively. Red arrow: ECs; yellow arrow: SCs (scale bar = 50 *μ*m).

We also examined how these scaffolds influenced the growth of ECs and SCs in culture (Figure 2). In Figure 2(a), TBO staining revealed that ECs (red arrow) acquired primarily cobblestone and oval morphologies after 3 days on scaffolds, while SCs (yellow arrow) mainly exhibited an elongated spindle-like morphology. Immunofluorescence staining for EC-specific platelet endothelial cell adhesion molecule-1 (CD31) and SC-specific S100*β* indicated that both cell types grew well on scaffold surfaces and that total cell numbers were significantly increased by inclusion of immobilized VEGF or IKVAV compared to bare CC scaffolds (*P* < 0.05) (Figure 2(b)) with no obvious difference between scaffolds immobilized with VEGF or IKVAV. However, CCHV scaffolds showed the highest fluorescence intensity for the EC marker CD31 compared to all other scaffolds, indicating that the heparin and VEGF most effectively facilitate EC proliferation (Figure 2(c)). Alternatively, there was no difference in the fluorescence intensity of S100*β* among biofunctionalized scaffolds (Figure 2(d)). The bare CC scaffolds exhibited the lowest fluorescence intensities for both CD31 and S100*β*, and CCHI scaffolds demonstrated significantly lower CD31 fluorescence intensity than all others. The results imply that single- and dual-biofunctionalized scaffolds support both EC and SC proliferation.

### 2.5. Expression of Angiogenic and Regenerative Genes by SCs and ECs on Dual-Biofunctionalized Scaffolds

**Figure 3:**
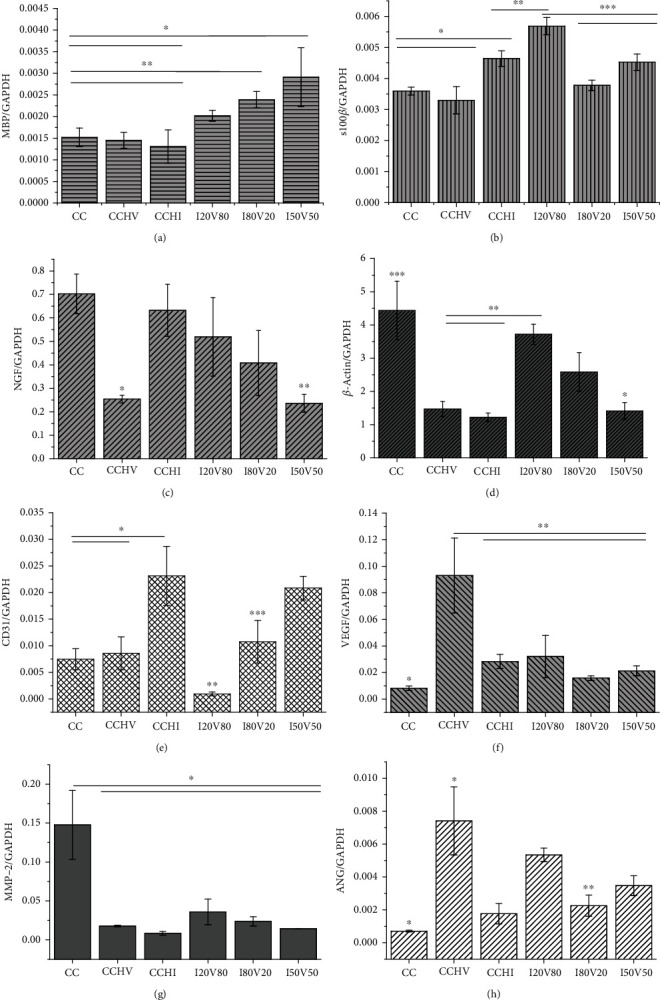
Gene expression analysis of ECs and SCs cultured in various scaffolds for 3 days: (a) MBP (myelin basic protein), (b) s100*β*, (c) NGF (nerve growth factor), (d) *β*-actin belonging to SCs, (e) CD31, (f) VEGF (vascular endothelial growth factor), (g) MMP-2 (matrix metalloproteinase), and (h) ANG (angiogenesis belonging to ECs). ^∗^*P* < 0.05, ^∗∗^*P* < 0.05, and ^∗∗∗^*P* < 0.05.

We then examined the expression levels of key angiogenic and regeneration-associated genes by PCR, including genes encoding myelin basic protein (MBP), S100*β*, nerve growth factor (NGF), and *β*-actin by SCs and CD31, VEGF, matrix metalloproteinase (MMP-2), and angiogenesis (ANG) by ECs. Compared to CC scaffolds, dual-biofunctionalized CCH scaffolds fabricated with different VEGF : IKVAV blending ratios significantly upregulated MBP expression by SCs, while no upregulation of MBP expression was observed on (single-biofunctionalized) CCHV or CCHI scaffolds (Figure 3(a)). In addition, I20V80 scaffolds markedly upregulated S100*β* expression by SCs compared to other scaffolds (Figure 3(b)). The bare CC scaffolds induced the greatest upregulation of NGF and *β*-actin expression (Figures 3(c) and 3(d)), whereas NGF and *β*-actin expression levels were lowest on CCHV scaffolds compared to all others. Notably, I20V80 scaffolds induced the greatest upregulation of NGF and *β*-actin expression by SCs among dual-biofunctionalized scaffolds. Both CCHI and I20V50 scaffolds induced high CD31 expression by ECs, while CCHV scaffolds induced the weakest CD31 expression (Figure 3(e)). In contrast, dual biofunctionalization did not upregulate the expression of MMP-2 compared to CC scaffolds (Figures 3(f) and 3(g)), while CCHV scaffolds significantly upregulated the expression of both VEGF and ANG by ECs (*P* < 0.05). The I20V80 and I50V50 scaffolds also induced greater ANG expression than pristine CC scaffolds. These results indicate that dual-biofunctionalized CC scaffolds can enhance the expression levels of angiogenic and neuroregeneration-associated genes in ECs and SCs.

### 2.6. Expression of Angiogenic and Regenerative Proteins by SCs and ECs on Dual-Biofunctionalized Scaffolds

**Figure 4:**
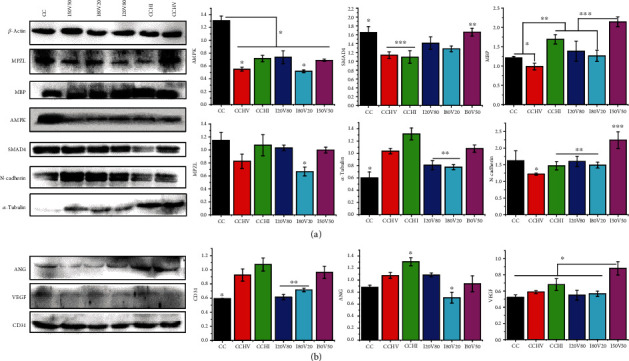
Protein expression analysis of ECs and SCs cultured in various scaffolds for 3 days using western blot: (a) protein expression in SCs including MPZL (myelin protein zero L), MBP, AMPK (adenosine 5′-monophosphate- (AMP) activated protein kinase), SMAD4 (drosophila mothers against decapentaplegic protein 4), N-cadherin, and *α*-tubulin and (b) protein expression in ECs including ANG, VEGF, and CD31. ^∗^*P* < 0.05, ^∗∗^*P* < 0.05, and ^∗∗∗^*P* < 0.05.

Changes in the expression levels of angiogenic and neuroregeneration-associated proteins by ECs and SCs growing on various scaffolds were measured using western blots. Compared to CC scaffolds, almost all biofunctionalized scaffolds downregulated the expression levels of adenosine 5′-monophosphate- (AMP) activated protein kinase (AMPK), myelin protein zero L (MPZL), and drosophila mothers against decapentaplegic protein 4 (SMAD4) with the exception of I50V50 scaffolds, which did not alter SMAD4 expression level compared to CC scaffolds (Figure 4(a)). The I50V50 scaffolds significantly upregulated the expression of *α*-tubulin by SCs compared to CC scaffolds. In addition, I50V50 scaffolds also induced the highest expression of MBP and N-cadherin by SCs compared to all other scaffolds. The CCHI scaffold induced the highest *α*-tubulin expression by SCs among all scaffolds, and all dual-biofunctionalized scaffolds induced greater SMAD4 expression than all single-biofunctionalized scaffolds. All biofunctionalized scaffolds also induced higher CD31 expression by ECs compared to CC scaffolds, with I50V50 scaffolds inducing higher CD31 expression than I20V80 and I80V20 scaffolds, while CCHI scaffolds induced the highest CD31 expression by ECs compared to all other scaffolds (Figure 4(b)). A similar pattern was observed for ANG expression by ECs except that I20V80 induced higher ANG expression than I80V20 and I50V50 scaffolds. However, I50V50 induced the greatest expression of VEGF by ECs compared to all other scaffolds. These results demonstrate that single- (VEGF or IKVAV) and dual-biofunctionalized scaffolds can differentially regulate the expression of angiogenic and neuroregeneration-associated proteins by SCs and ECs.

### 2.7. Evaluation of Vascularization Induction by Dual-Biofunctionalized Scaffolds

**Figure 5:**
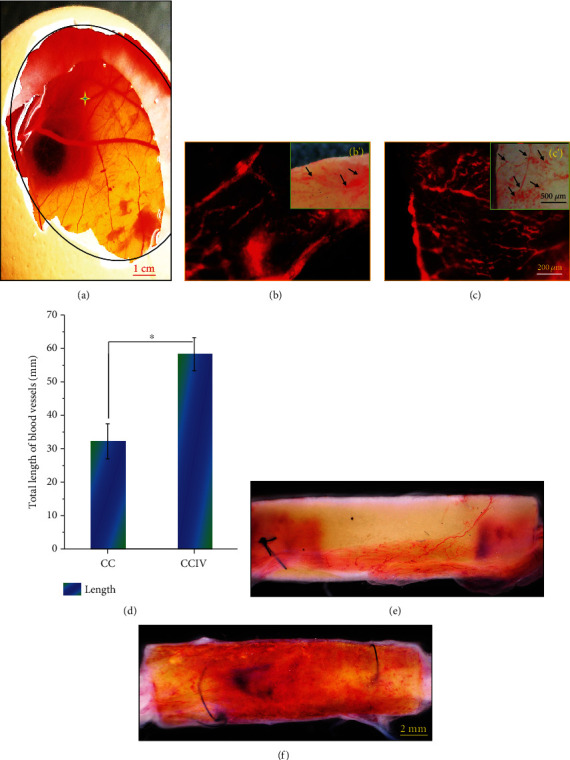
Vascularization evaluation of chitosan/collagen (CC) scaffolds and dual-biofunctionalized scaffolds with VEGF/IKVAV (CCIV) using (a–d) chicken embryo experiment for 8 days and(e, f) sciatic nerve implantation for 14 days. (a) Implantation site of scaffolds in chicken embryos, (b, c, b', c') CD31 staining and optical images of newborn blood vessels (scale bar = 200 *μ*m), (d) total length of blood vessels in different scaffolds, and vascularization effect of (e) CC scaffolds and (f) CCIV scaffolds in an injured rat sciatic nerve (scale bar = 2 mm).

The vascularization induction potentials of CC and I50V50 (CCIV) scaffolds were evaluated by implanting these constructs into chicken embryos for 8 days. The cross-star in Figure 5(a) shows the implantation location of the scaffolds. Fluorescence analysis of CD31 labeling revealed that CCIV scaffolds induced the development of complex capillary networks with numerous branches, while few vessel branches were observed around implanted CC scaffolds. The optical microscope images of vascularization in different scaffolds in Figures 5(b') and 5(c')further verified the above results. In addition, total blood vessel length was greater around CCIV scaffolds than CC scaffolds in Figure 5(d). Obviously, CCIV scaffolds possessed significantly longer blood vessels than CC scaffolds. Besides implantation into chicken embryos, both scaffolds were also implanted into the injured site of a rat sciatic nerve to evaluate the vascularization effect (Figures 5(e) and 5(f)). In accordance with the results in chicken embryos, numerous blood vessels grew on both inner compartments and surfaces of dual-biofunctionalized scaffolds, while few blood vessels were observed on the surfaces and within CC scaffolds. Thus, dual-biofunctionalized VEGF/IKVAV scaffolds were more effective than single-biofunctionalized scaffolds for promoting the vascularization of nerve implants.

### 2.8. Early-Stage Sciatic Nerve Regeneration Induced by Dual-Biofunctionalized Scaffolds

**Figure 6:**
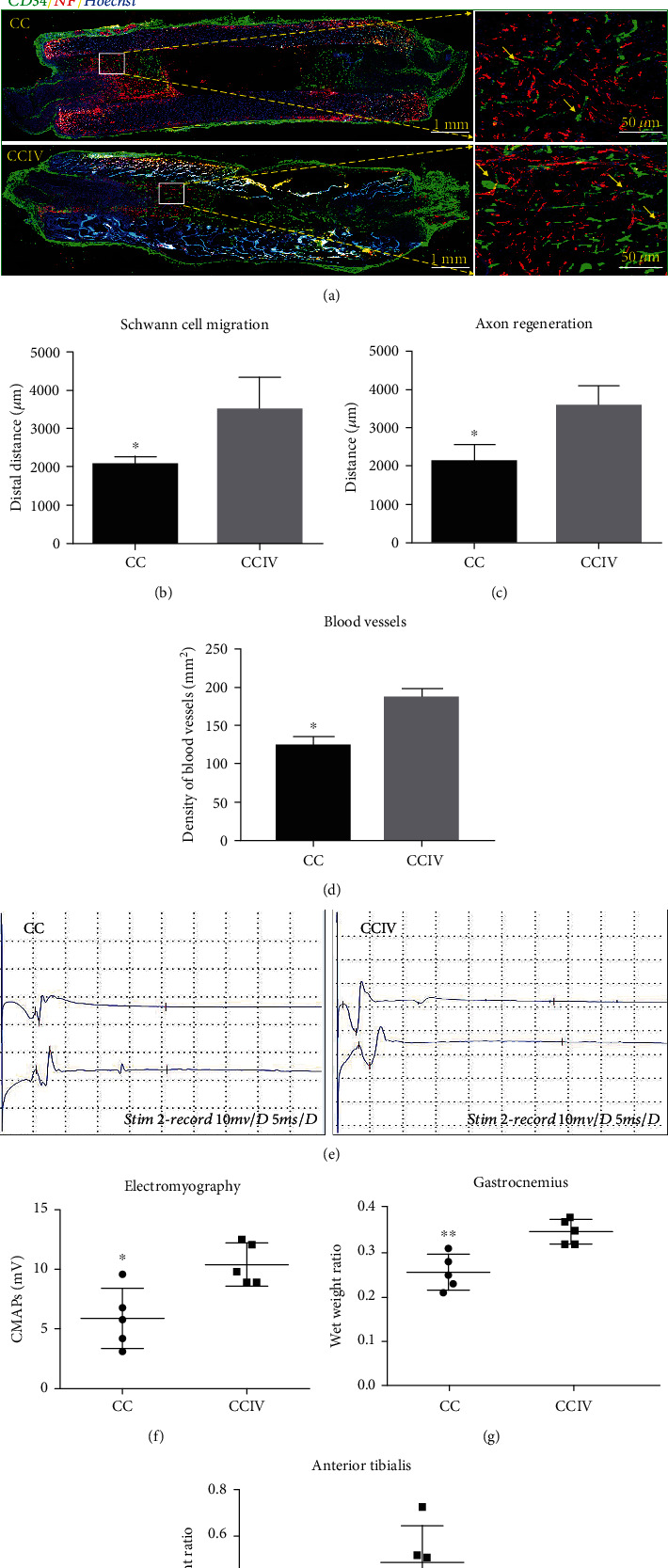
Evaluation of the rat sciatic nerve using immunohistochemistry after scaffold implantation for 14 days. (a) Immunohistochemistry staining of NF200 (neurofilament200, red), CD34 (hematopoietic progenitor cell antigen, green), s100*β* (red), and Hoechst 33342 (blue) of the longitudinal sections of the regenerating nerve harvested at 14 d after bridging a 10 mm rat sciatic nerve gap with a plain CC scaffold and a dual-biofunctionalized scaffold with IKVAV/VEGF, respectively. The right images are the magnifications of the boxed areas. Left scale bar = 1 mm and right scale bar = 50 *μ*m. (b–d) Histograms comparing (b) the migration of SCs, (c) the length of regenerating nerve fibers, and (d) the density of blood vessels regenerated in the scaffolds at 14 d after surgery (^∗^*P* < 0.05). And (e, f) comparison of compound muscle action potential (CMAP) amplitudes and (g, h) the wet weight ratio of the gastrocnemius and anterior tibialis muscles. Data are expressed as means ± SD. ^∗^*P* < 0.05 versus control group.

The immunohistochemistry staining for neurofilament 200 (NF200) (red), hematopoietic progenitor cell antigen (CD34) (green), and S100*β* (red) plus Hoechst 33342 nuclear counterstaining (blue) was performed to evaluate axonal regrowth and vascularization in a longitudinally sectioned rat sciatic nerve 14 days postinjury, respectively.  Figure 6(a) shows that the regenerating axons elongated from the proximal stump toward the distal stump. But the gap between proximal and distal ends was smaller using CCIV scaffold implants than CC implants. Statistical analysis in Figures 6(b) and 6(c) further confirmed that the migration of SCs and the lengths of regenerating axons were significantly enhanced in nerves implanted with CCIV scaffolds compared to CC scaffolds at this early stage of regeneration (*P* < 0.05). In addition, the density of newly regenerated blood vessels was greater in nerves implanted with CCIV scaffolds than those implanted with CC scaffolds as evidenced by CD31 antibody labeling (Figure 6(d), yellow arrow). These results demonstrate that scaffolds biofunctionalized with IKVAV and VEGF accelerate SC migration, axonal elongation, and angiogenesis following peripheral nerve injury. Further, these dual-biofunctionalized scaffolds also enhanced functional recovery as evidenced by electrophysiological measures of neuromuscular transmission and muscle wet weight ratio at 14 days postinjury (Figures 6(e)–6(h)). The compound muscle action potentials (CMAPs) evoked by stimulation of the distal or proximal end of the injured nerve were significantly larger in rats implanted with CCIV scaffolds (~11 mV) compared to CC scaffolds (~6 mV) (Figures 6(e) and 6(f)). In addition, wet weights of the target anterior tibial and gastrocnemius muscles from the injured side were significantly larger in rats implanted with CCIV scaffolds compared to CC scaffolds, suggesting enhanced target reinnervation by the sciatic nerve, while wet weights did not differ on the uninjured side (Figures 6(g) and 6(h)).

### 2.9. Late-Stage Sciatic Nerve Regeneration Induced by Dual-Biofunctionalized Scaffolds

**Figure 7:**
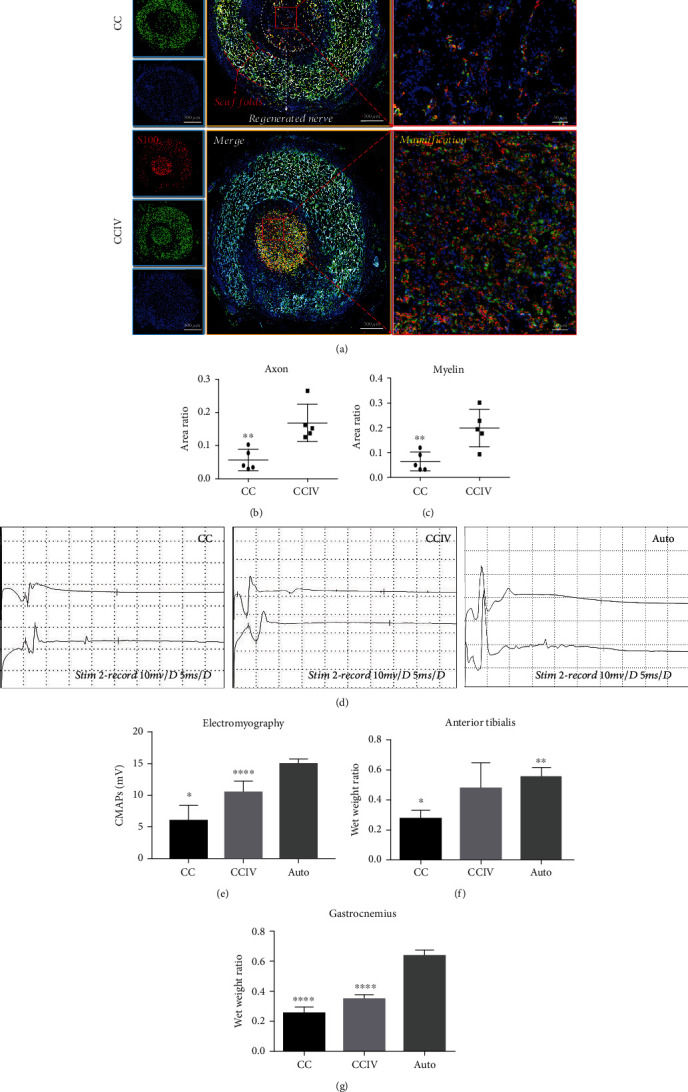
Evaluation of sciatic nerve regeneration after 12 w of surgery. (a) Immunofluorescence staining with an anti-NF200 antibody, anti-S100 antibody, and Hoechst 33342 for the nerve fiber, myelination, and nucleus. (b, c) Area ratio of the regenerated axon and myelin (^∗∗^*P* < 0.05). (d, e) Electrophysiological assessment of the regenerated sciatic nerve (^∗^*P* < 0.05: CC group versus CCIV and Auto groups, ^∗∗∗∗^*P* < 0.05: CCIV versus Auto groups). (f, g) Wet weight ratio of the anterior tibialis and gastrocnemius muscles (^∗^*P* < 0.05: CC group versus CCIV and Auto groups, ^∗∗^*P* > 0.05: CCIV versus Auto groups, and ^∗∗∗∗^*P* < 0.05: CC and CCIV groups versus and Auto group).

Immunofluorescence staining for NF200 and S100 plus Hoechst 33342 nuclear counterstaining was also conducted on transverse sciatic nerve sections to evaluate axonal regeneration and myelination (Figures 7(a)–7(c)) 12 weeks postinjury. The density of regenerated nerve fibers (red and green) was markedly greater in nerves implanted with CCIV scaffolds compared to CC scaffolds (Figure 7(a)). Further, the area ratios of axons (Figure 7(b)) and myelin (Figure 7(c)) were significantly higher in nerves implanted with CCIV scaffolds compared to CC scaffolds (axon: 0.18 ± 0.02 vs. 0.05 ± 0.003; myelin: 0.21 ± 0.04 vs. 0.04 ± 0.001, both *P* < 0.05). Direct comparison of CMAP amplitudes among CC-implanted, CCIV-implanted, and autologous (Auto) groups (Figures 7(d) and 7(e)) indicated that sciatic neuromuscular transmission was significantly greater in rats implanted with CCIV scaffolds compared to CC scaffolds (11.12 ± 1.41 vs. 6.51 ± 1.53 mV, *P* < 0.05) although these CAMPs were still smaller compared to the autologous group (15.06 ± 1.08 mV, both *P* < 0.05). The wet weight ratios of target anterior tibialis and gastrocnemius muscles are shown in Figures 7(f) and 7(g). Implantation of CCIV scaffolds also resulted in significantly greater wet weight ratios of the anterior tibialis and gastrocnemius muscles compared to CC scaffold implantation (both *P* < 0.05). Further, although the weight ratio of the gastrocnemius muscles was still smaller in the CCIV group than in the Auto group (*P* < 0.05), the wet weight ratio of the anterior tibialis did not differ between CCIV and Auto groups (*P* > 0.05). Thus, CCIV scaffolds enhanced regeneration and neuromuscular transmission of the sciatic nerve compared to CC scaffolds.

### 2.10. TEM Analysis of the Myelin Sheath

**Figure 8:**
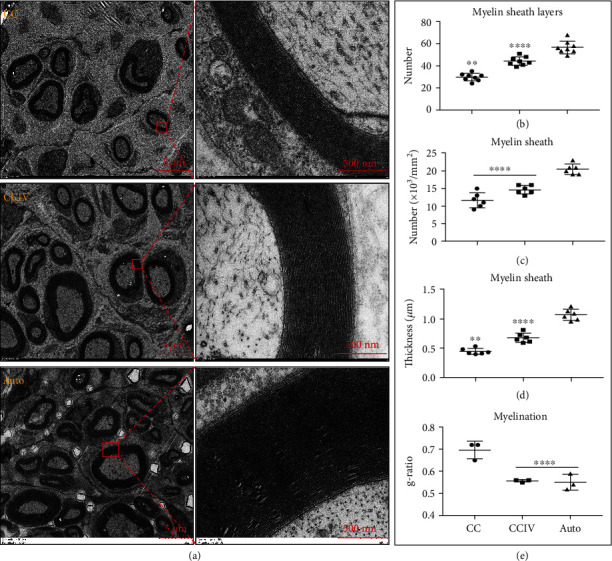
Observation of regenerated nerve fibers using a transmission electron microscope (TEM) at 12 weeks after surgery for rats in CC (chitosan/collagen scaffolds), CCIV (chitosan/collagen scaffolds with loaded IKVAV and VEGF), and Auto groups. (a) Regenerated nerves at the 5 mm sites of the distal sciatic nerve: right images are the magnification of the left red box. (b) Number of myelin sheath layers (^∗∗^*P* < 0.05: CC group versus CCIV and Auto groups, ^∗∗∗∗^*P* < 0.05: CCIV versus Auto groups). (c) Number of myelin sheaths (^∗∗∗∗^*P* < 0.05: CC and CCIV groups versus Auto group). (d) Thickness of myelin sheaths (^∗∗^*P* < 0.05: CC group versus CCIV and Auto groups, ^∗∗∗∗^*P* < 0.05: CCIV versus Auto groups). (e) g-ratio of myelination of the regenerated nerve (^∗∗∗∗^*P* < 0.05: CC group versus CCIV and Auto groups).

Finally, the ultra-microstructures of distal nerve segments in CC, CCIV, and Auto groups were examined by transmission electron microscopy (TEM) (Figure 8). Regenerated myelinated fibers demonstrated a uniform circular structure, with smooth SC basal membranes and electron-dense myelin sheaths in all the three groups (Figure 8(a)). However, the total number of myelin sheath layers was significantly greater in the Auto and CCIV groups compared to the CC group (58 ± 5.8 and 42 ± 2.6 vs. 31 ± 3.5, both *P* < 0.05) (Figure 8(b)). Similarly, both the mean number (Figure 8(c)) and mean thickness (Figure 8(d)) of myelin sheaths were highest in the Auto group followed by the CCIV group and smallest in the CC group. Consistent with these findings, the g-ratio was highest in the CC group (> 0.7) but did not differ between Auto and CCIV groups (<0.6) (Figure 8(e)). These results further demonstrate that CCIV scaffolds can effectively improve myelination compared to CC scaffolds (although they do not completely restore myelination to preinjury levels).

## 3. Discussion

Despite the considerable success of artificial nerve implants for improving peripheral nerve regeneration [33, 34], this treatment is still beset by limitations including the lack of effective induction cues for axon growth and Schwann cell migration and poor nutrient supply due to insufficient neovascularization, ultimately resulting in incomplete functional recovery. These drawbacks have inspired the development of numerous implantable scaffolds with physiochemical and/or biological premodifications designed to enhance bridging of the injured nerve [35]. To date, proteins and biomolecules such as fibronectin, IKVAV, and Tyr-Ile-Gly-Ser-Arg (YIGSR) have been incorporated into scaffolds to promote nerve cell adhesion and axon growth, and indeed these constructs have proven partially successful. In addition, vascular induction has been improved by immobilizing angiogenesis-related biofactors such as VEGF and platelet-derived growth factor (PDGF) [36]. However, few studies have examined combinations of bioactive factors for simultaneously promoting nerve regrowth and vascularization. Therefore, dual-biofunctionalized chitosan/collagen scaffolds with immobilized VEGF and IKVAV were developed and systematically evaluated for physicochemical properties, *in vitro* capacity to promote SC and EC proliferation and survival, and efficacy for restoring myelination, axonal integrity, and neuromuscular transmission. These biofunctional scaffolds enhanced SC and EC proliferation rates, the expression levels of angiogenic and neuroregeneration-related genes and proteins by these cells, and neovascularization, axonal repair, myelination, and neuromuscular transmission *in vivo* compared to bare CC scaffolds and biofunctionalized scaffolds incorporating only IKVAV or VEGF. Chitosan is a highly cationic polysaccharide [37], while collagen mainly carries anionic groups [38], thus facilitating strong CC complex formation via electrostatic interactions. After confirming this complex formation by UV spectroscopy, dual-biofunctionalized chitosan/collagen composite scaffolds were constructed by immobilizing IKVAV and VEGF via heparin as confirmed by various quantitative and qualitative analyses including FTIR, TBO staining, ELISA, and fluorescence staining. The IKVAV sequence in laminin mediates specific binding to the laminin receptor, which in turn promotes neuronal adhesion and neurite outgrowth [25]. Moreover, IKVAV was also found to induce endothelial cell tube formation, angiogenesis, and revascularization [39, 40]. Similarly, VEGF is a critical mediator of angiogenesis. Numerous methods, including chemical cross-linking and physical adsorption, have been used for immobilizing biomolecules on biomaterial surfaces. However, chemical cross-linking may shield the bioactive site, thereby reducing functional activity, while adsorption may induce biomolecule instability. In this study, heparin was used to immobilize IKVAV and VEGF in the composite scaffolds through peptide and electrostatic interactions. Heparin is a negatively charged biomolecule with cationic groups on the side chains allowing electrostatic binding to chitosan without influencing the activity of other bound biomolecules. Generally, scaffolds with directly immobilized bioactive factors show burst-like release once implanted *in vivo*. However, heparin can combine with VEGF and IKVAV via specific peptide-peptide interactions and electrostatic attractions, respectively, resulting in sustained release over the course of nerve repair [41]. Indeed, heparin has been shown to maintain the bioactivity and sustained release of growth factors [42] and so was chosen as the carrier for simultaneous delivery of IKVAV and VEGF in this study. In accordance with previous findings, heparin coating resulted in the successful immobilization and stable release of VEGF and IKVAV.

The *in vitro* coculture of Schwann cells and endothelial cells was then performed to evaluate the biocompatibility of the prepared scaffolds. The biofunctionalized scaffolds in this study permitted better cell growth than heparinized scaffolds. Schwann cells promote axon outgrowth and myelination by secreting various neurotrophic factors such as NGF and brain-derived neurotrophic factor (BDNF) as well as extracellular matrix components that provide a growth-permissive substrate for peripheral nerve regeneration [43], while ECs are an important component of capillaries and thus help provide nutrients necessary for axon and SC growth [44]. Further, SCs and ECs communicate via chemical signaling. For instance, VEGF-related factors derived from SCs may induce arterial differentiation from endothelial cell precursors and promote specific vascular patterns [45], whereas nitric oxide secreted by endothelial cells was reported to suppress Schwann cell dedifferentiation [44]. Therefore, the cogrowth of Schwann cells and endothelial cells is anticipated to simultaneously promote neovascularization and nerve regeneration, and our dual-biofunctionalized scaffolds indeed provided a platform conducive to the growth and differentiation of both cell types. These dual-biofunctionalized scaffolds (especially I50V50) also effectively upregulated MBP and CD31 expression by these cells, possibly through the activation of the PI3K-CREB pathway [46] and PKA signal pathway [47], respectively. MBP and CD31 are both critical markers of early-stage myelination [48] and vascularization, respectively, underscoring the potential utility of these dual-biofunctionalized scaffolds for simultaneous induction of myelination and vascularization. In addition, western blotting indicated that I50V50 scaffolds more effectively upregulated the expression levels of SMAD4, MBP, *α*-tubulin, N-cadherin, CD31, and VEGF, factors also critical for promoting vascularization and nerve regeneration, compared to other scaffolds. Moreover, upregulation of MBP and CD31 at the mRNA level by I50V50 scaffolds was confirmed by PCR. Based on these findings, the I50V50 scaffold was chosen for further evaluation of vascularization and nerve regeneration *in vivo*.

Rapid and extensive neovascularization is required to ensure the long-term survival and tissue integration of implants [49]. These newly formed blood vessels are required to provide nutrient substances necessary for Schwann cell differentiation and axon growth. In the present study, VEGF was used to enhance angiogenesis via surface cross-linking to heparin as previously reported using polycaprolactone scaffolds [42]. These dual-biofunctionalized scaffolds effectively promoted neovascularization and increased blood vessel branching and elongation within days to weeks in chicken embryos and the rat sciatic nerve injury model, further confirming that IKVAV/VEGF dual-biofunctionalized scaffolds may be a promising choice for restoring blood circulation to support the early stage of regeneration. This rapid induction also suggests the potential for extending CCIV use to other various tissue engineering applications. In the current study, implantation for 12 w promoted some functional recovery as revealed by partial restoration of compound muscle action potential (CMAP) amplitude, target muscle wet weight ratio, and sciatic nerve axonal and myelin sheath microstructure. Functional regeneration was significantly better using IKVAV/VEGF dual-biofunctionalized chitosan/collagen scaffolds compared to the bare CC scaffold.

To the best of our knowledge, this is the first study to describe the construction of IKVAV/VEGF dual-biofunctionalized chitosan/collagen scaffolds and demonstrate the capacity of these constructs to both enhance neovascularization and neuroregeneration of peripheral nerves. The present study may thus provide a new strategy for regenerating vascularized tissue. However, a more effective dual release system that adapts to the current stage of regeneration may further improve the functional outcome. The effects of these scaffolds on SCs and ECs in different proportions and on the interactions between these cells also warrant further study. Finally, the detailed mechanisms and signal pathways activated by these dual-biofunctionalized scaffolds during microvascular formation and nerve regeneration remain poorly understood, and such knowledge could help in the further refinement of these constructs.

## 4. Conclusion

In summary, dual-biofunctionalized chitosan/collagen scaffolds with IKVAV/VEGF were successfully constructed by combining lyophilization and surface modification. The prepared scaffolds enhanced the attachment, proliferation, and survival of Schwann cells and endothelial cells. In addition, IKVAV/VEGF dual-biofunctionalized chitosan/collagen (I50V50) scaffolds upregulated myelination- and vascularization-related genes (MBP, CD31) and proteins (SMAD4, MBP, *α*-tubulin, N-cadherin, CD31, and VEGF) *in vitro*, enhanced vascularization in chick embryos, and promoted neovascularization, axonal regeneration, myelination, and neuromuscular transmission following rat sciatic nerve injury. The results highlight the potential of dual-biofunctionalized chitosan/collagen scaffolds for improving peripheral nerve regeneration in the clinic. Such a strategy may provide a critical experimental and theoretical reference for designing and developing the new generation of artificial implants for nerve regeneration.

## Supplementary Materials

Supplementary Materials Supplementary Materials 1 Experimental section and figure legends. Figure S1: sketch map of the preparation of dual-biofunctionalized chitosan/collagen composite scaffolds. Figure S2: characterization of scaffold preparation. (A) Ultraviolet (UV) spectra of collagen (CO), chitosan (CS), and chitosan/collagen (CC), (B) toluidine blue (TBO) staining, (C) distribution of heparin, and (D) heparin amount (^∗^*P* < 0.05 versus other samples). Figure S3: immobilization amount and release behavior of vascular endothelial growth factor (VEGF) (A-C) and Ile-Lys-Val-Ala-Val (IKVAV) (D-F) in various scaffolds. ^∗^*P* < 0.05, ^∗∗^*P* < 0.05 versus other samples.
