# Supplementary material for: Construction of Dual-Biofunctionalized Chitosan/Collagen Scaffolds for Simultaneous Neovascularization and Nerve Regeneration
Source: Research (Wash D C). 2020 Aug 10;2020:2603048. doi: 10.34133/2020/2603048 (PMC7436332; doi:10.34133/2020/2603048)
Supplement: Supplementary Materials — 1 Experimental section and figure legends. Figure S1: sketch map of the preparation of dual-biofunctionalized chitosan/collagen composite scaffolds. Figure S2: characterization of scaffold preparation. (A) Ultraviolet (UV) spectra of collagen (CO), chitosan (CS), and chitosan/collagen (CC), (B) toluidine blue (TBO) staining, (C) distribution of heparin, and (D) heparin amount (∗P < 0.05 versus other samples). Figure S3: immobilization amount and release behavior of vascular endothelial growth factor (VEGF) (A-C) and Ile-Lys-Val-Ala-Val (IKVAV) (D-F) in various scaffolds. ∗P < 0.05, ∗∗P < 0.05 versus other samples. [file 2603048.f1.doc]

Title

Construction of Dual-Biofunctionalized Chitosan/Collagen Scaffolds for Simultaneous Neovascularization and Nerve Regeneration

**Authors**

Guicai Li ^1,2*^, Qi Han ^1,2^, Panjian Lu ^1,2^, Liling Zhang ^1,2^, Yuezhou Zhang^3^, Shiyu Chen ^1,2^, Ping Zhang^1,2^, Luzhong Zhang ^1,2^, Wenguo Cui^4^, Hongkui Wang ^1,2*^, Hongbo Zhang^4,5*^

**Affiliations**

^1^Key laboratory of Neuroregeneration of Jiangsu and Ministry of Education, Nantong University, 226001, Nantong, P.R. China

^2^Co-innovation Center of Neuroregeneration, Nantong University, 226001, Nantong, P.R. China

^3^Xi’an Institute of Flexible Electronics & Xi’an Institute of Biomedical Materials and Engineering, Northwestern Polytechnical University, Xi’an 710072, China.

^4^Shanghai Institute of Traumatology and Orthopaedics, Shanghai Key Laboratory for Prevention and Treatment of Bone and Joint Diseases, Ruijin Hospital, Shanghai Jiao Tong University School of Medicine, 197 Ruijin 2nd Road, Shanghai 200025, PR China

^5^Pharmaceutical Science Laboratory and Turku Bioscience Center, Åbo Akademi University, 20520, Turku, Finland

Correspondence should be addressed to Guicai Li; [gcli1981@ntu.edu.cn](mailto:gcli1981@ntu.edu.cn), Hongkui Wang; [wanghongkui@ntu.edu.cn](mailto:wanghongkui@ntu.edu.cn), and Hongbao Zhang; [hongbo.zhang@abo.fi](mailto:hongbo.zhang@abo.fi)

**1 Experimental section**

**1.1 Materials and reagents**

Chitosan with an average molecular weight of 100 kDa and deacetylation of 92% was purchased from Nantong Xincheng Chemicals Co., Ltd., Jiangsu, PR China. Collagen sheet from bovine tendon (Type I) was bought from Sannie Bioengineering Technology CO., LTD. Acetic acid was purchased from J&K scientific Co., Ltd., Shanghai, PR China. Dopamine hydrochloride, VEGF, forskolin, anti-Thy 1.1 antibody, polyvinylidene fluoride (PVDF) membrane were all bought from Sigma-Aldrich, USA. Ile-Lys-Val-Ala-Val (IKVAV) and FITC labelled IKVAV were bought from Shanghai Jill Biochemical Co. Ltd., China, Glutaraldehyde was purchased from Hengchang Chemical Co., Ltd., Nantong, PR China. Paraformaldehyde, glutaraldehyde were from National Pharmaceutical Group Chemical Reagent Co. Ltd., China. Type I collagenase, penicillin, streptomycin, Dulbecco’s modified Eagle’s medium (DMEM), M199 and 0.25% trypsin-EDTA were all purchased from Invitrogen Co., Ltd.,Carlsbad, CA). Mouse anti-human CD31 antibody, rabbit anti-rat s100β antibody and DAPI were all purchased from Sigma-Aldrich, USA. Rabbit anti-rat VEGF antibody, Rabbit anti-rat NF200 antibody was bought from Boosen, China, TRITC-goat anti-rabbit IgG and FITC-goat anti-mouse IgG were both bought from Boster Biological Technology Co. Ltd., USA. Toluidine Blue, TBST buffer, VEGF ELISA kit, BCA kit, sheep serum and heat-inactivated fetal bovine serum (FBS) was purchased from 4A Biotech Co., Ltd., CN. Primers for RT-PCR test were designed by Livsing, China. Total RNA from the cell lysate was extracted using a Trizol kit from Invitrogen, CA. cDNA from total RNA was synthesized using an Omniscript RT kit from Qiagen, CA. The distilled water (dH_2_O) was used for all processes. Sodium hydrate (NaOH), hydrochloric acid (HCl), ethanol and other reagents used in this study were all A.R. grade reagents without any further purification.

**1.2 Fabrication of dual-biofunctionalized chitosan/collagen scaffolds and conduits**

The chitosan/collagen composite scaffolds were prepared as follows (Figure.S1): Firstly, a 1wt.% chitosan solution and a 1wt.% collagen solution was separately obtained by dissolving chitosan powder or collagen sheet in 1% acetic acid aqueous solution under gentle shaking at room temperature. Then both solutions were mixed thoroughly with the volume proportions of 1:1, and permitted to store statically for 2 h to remove the trapped air bubbles. Thereafter, the mixed solution was poured into a 24-well cell culture dish and lyophilized at -50 ℃ in a freeze dryer (Virtis Wizard, US) for 24 h to form chitosan/collagen scaffolds (CC). After that, the formed scaffolds were treated with a 0.1 M NaOH aqueous solution for 2 h to neutralize the residual acetic acid. And then, the scaffolds were rinsed with dH_2_O for 10 times until reaching a neutral status. Subsequently, the scaffolds were lyophilized again at -50°C for 24 h and stored in dehumidifying device before further use. The pure chitosan (CS), collagen (CO) scaffolds was also prepared by the same method and used as control in the study.

The preparation of dual-biofunctionalized chitosan/collagen scaffolds was described as following: A heparin solution with concentration of 2mg/mL in PBS was firstly prepared, and then the chitosan/collagen scaffolds were immersed into heparin solution at RT for 6 h to obtain the heparinized-chitosan/collagen (CCH) scaffolds. After that, the above scaffolds were rinsed with PBS for three times to remove the residual heparin. Secondly, VEGF (100ng) and IKVAV (100 μg/mL) solution diluted in PBS was blended with different volume proportions (100:0, 80:20, 50:50, 20:80, 0:100) to prepare VEGF/IKVAV mixture. Then, the CCH scaffolds with diameter of 10 mm were placed into a 24-well culture plate and incubated with 200 μL VEGF/IKVAV mixture for 120 min at room temperature. After that, the scaffolds were rinsed with PBS for three times, each for 5min, and then stored at -4℃ with humidity of 45% for use. Simultaneously, the CCH scaffolds modified with single VEGF or IKVAV were used as control. The name of the biofunctionalized CCH scaffolds was named as CCHV, I20V80, I50V50, I80V20, CCHI according to the volume proportions of VEGF and IKVAV. In addition, for immunofluorescence observation, the FITC-labelled IKVAV was also immobilized on the scaffolds using the same method. The optimal parameter of VEGF and IKVAV ratio was used for future biofunctionalizing chitosan/collagen conduit and animal experiment.

For preparing dual-biofunctionalized and bare chitosan/collagen conduit, the blending solution was firstly injected into a home-made mold and then lyophilized at -50 ℃ in a freeze dryer for 24 h. After that, the conduit was peeled off and treated using the same method mentioned above to obtain the bare chitosan/collagen conduit and VEGF/IKVAV immobilized chitosan/collagen conduit.

**1.3 Characterization of scaffolds**

**1.3.1 UV spectra**

The single CS, CO and CS solution with volume of 1mL were separately loaded into a quartz tube and mounted onto the measurement chamber. The UV spectra of standard and sample solutions were then recorded by a spectrophotometer (Jinghua, Shanghai, CN).

**1.3.2 Quantity and quality analysis of heparin**

The quantitative and qualitative analysis of heparin in chitosan/collagen composite scaffolds were referring to our previously study[[1](#_ENREF_1)] by TBO assay and modified here. Briefly, the scaffolds were firstly immersed into an aqueous TBO solution (0.04 wt%,

5 mL) in 0.01 M HC1/0.2 wt% NaC1, and gently shaken at 37 ◦C for 4 h. For qualitative analysis of heparin, the scaffolds above were washed for five times with dH2O (each for 10 min), and observed using an optical microscope (Leica, Germany). For quantitative analysis of heparin, the above scaffolds were firstly immersed into a mixture (4/1, 5 mL) of ethanol and aqueous 0.1 M NaOH for 10 min, then the supernatant (200 μL) was transferred into a 96-well plate, and the absorbance at 530 nm was recorded by a microplate reader (Bio Tek, USA). The quantity of heparin was calculated via the calibration curve.

**1.3.3 FTIR spectra**

A Fourier transform infrared spectrometer (FTIR, Nicolet5700, Madison, WI) with potassium bromide tablets and transmission mode was used to detect the component variation of the scaffolds as a function of biomodification using VEGF and IKVAV. The infrared absorption spectra were obtained after scanning in the range from 400 to 4500 cm^-1^. A total of 64 scans were accumulated at 4 cm^-1^ resolution for each spectrum.

**1.3.4 SEM observation**

A scanning electron microscopy (SEM, Hitachi S-3400 NII, Japan) was used to observe the surface morphology of the prepared scaffolds. Briefly, the scaffolds were dried and fixed to an aluminum stage using a double-sided conductive tape, and then coated with a layer of gold with thickness of 50 nm. Then, the coated scaffolds were observed by SEM under the vacuum degree of 1.5×10^-4^Pa. Seven randomly selected sight fields were captured under the same magnification and analyzed for each sample.

**1.3.5 AMF observation**

AFM (Nanowizard II, JPK Instruments, Berlin, Germany) was further used to observe the surface topography and roughness of all the treated scaffolds using an in tapping mode. The dried scaffolds were mounted onto the measurement platform of AFM. And all the tests were performed at a scan rate of 0.5 Hz using Si cantilevers at room temperature. A CSPM Imager software was used to perform image analysis to obtain the surface morphology and roughness of various scaffolds.

**1.4 Determination of the loaded VEGF and IKVAV**

For VEGF, an indirect ELISA method was used to determine the amount of loaded VEGF in various scaffolds. Briefly, the scaffolds (diameter 10mm) were placed into a 24-well culture plate and blocked with sheep serum (1 wt% inPBS) at 37 ◦C for 30 min. Then, the rabbit monoclonal anti-rats VEGF antibody (20 μL, diluted 1:250 in PBS) was added and incubated for 2 h at 37 ◦C. After that, the scaffolds were thoroughly washed with PBS for five times, and the HRP-labeled sheep polyclonal anti-rabbit IgG antibody (20 μL, diluted 1:100 in PBS) was added and incubated at 37 ◦C for another 2 h. Subsequently, the scaffolds were thoroughly rinsed with PBS for five times, and 100μL TMB solution was added onto the scaffolds and kept in dark for 10 min. Furtherly, 100μL H_2_SO_4_ (1 M) was added to stop the peroxidase catalyzed reaction. Finally, 150μL supernatant was transferred to a 96-well plate to detect the absorbance at 450 nm with a microplate reader. The amount of loaded VEGF was calculated according to the calibration curve. In addition, a similar method was used to detect the distribution of VEGF on various scaffolds. But the difference was that a FITC-labelled sheep polyclonal anti-rabbit IgG antibody was used, and finally the scaffolds was observed by fluorescence microscope (Leica, Germany).

For IKVAV, a BCA kit was used to determine the amount of IKVAV according to the inserted instruction. In brief, the scaffolds were firstly treated with the same method as mentioned above. Then, 500μL BCA solution was added to each well and incubated at 37 ◦C for 30 min under gentle shaking. After that, 200μL supernatant from each well was transferred to a 96-well plate, and the absorbance at 562 nm was measured. The amount of loaded IKVAV was calculated according to the calibration curve. To detect the distribution of IKVAV on various scaffolds, the FITC-labelled IKVAV was firstly immobilized onto the scaffolds at 37 ◦C for 2 h, and then the scaffolds was rinsed for three times and directly observed by fluorescence microscope

**1.5 Release of VEGF and IKVAV**

The release profile of loaded VEGF and IKVAV from different scaffolds was determined using VEGF ELISA kit and BCA kit according to the inserted package instruction, respectively. The VEGF/IKVAV loaded scaffolds (10 × 10 mm) were placed into a 24-well culture plate, and 2mL PBS was added and incubated at 37 ◦C for different periods (from 0 to 50 h). Then, the supernatant at each time point was harvested and the release of VEGF and IKVAV into supernatant was detected using the corresponding kit.

**1.6 Co-culture of SCs and ECs**

Schwann cells were isolated from Sprague-Dawley rat pups (2–5 day old) and cultured until confluency according to our previous study[]. The third passage of Schwann cells was used in this experiment. Endothelial cells (ECV304) was bought from Shanghai Cell Bank, China. For co-culture of ECs and SCs, the sterile scaffolds (10 × 10 mm) with VEGF/IKVAV immobilization were firstly placed into a 24-well cell culture plate. Then, both cells were trypsinized with trypsin in PBS buffer when cells approached confluence. After centrifugation and resuspension in culture medium, SCs with a concentration of 1x10^5^ cells/mL in DMEM and ECs with a concentration of 5x10^4^ cells/mL M199 were mixed gently at a volume ratio of 1:1. Subsequently, 1 mL of the mixed cell suspension was taken and added onto each scaffolds. Finally, cells were co-incubated with scaffolds for 3 days before further evaluation.

**1.7 Morphology and quantity evaluation of cells**

The morphology of SCs and ECs after culture on scaffolds for 3 days was evaluated using immunofluorescence staining. Briefly, the scaffolds containing both cells were thoroughly rinsed for five times with PBS and then fixed with paraformaldehyde (4 wt.%) for 1h. Then, the scaffolds were washed with PBS and blocked with 1% sheep serum at 37 ◦C for 30 min. Afterwards, the mouse anti-human CD 31 antibody (1:250 diluted in PBS) and rabbit anti-rat s100β antibody (1:250 diluted in PBS) with 50 μL of each was added to the scaffolds and incubated at 37 ◦C for 2h. Then, the scaffolds were rinsed with PBS for three times and TRITC-goat anti-rabbit IgG (1:100 diluted in PBS) and FITC-goat anti-mouse IgG antibody(1:100 diluted in PBS) with 50 μL of each was added and incubated at 37 ◦C for 2h. After further rinse with PBS, 50 μL DAPI (5μg/mL) was added onto each scaffolds and incubated at 37 ◦C for 30 min. Finally, all scaffolds containing cells were observed under a fluorescence microscope. Notably, the whole experiment was performed in darkness. In addition, the intensity of fluorescence was also measured to evaluate the cell number of SCs and ECs, respectively. For quantitative evaluation of both cells on scaffolds after 3 d of culture, a CCK-8 kit was used according to the inserted instruction in the package. In brief, the culture medium at 3 d was removed and the fresh prepared culture medium containing CCK-8 reagent (1:9 in medium) was added and incubated at 37 ◦C for 4 h. Subsequently, 200 μL of supernatant was transferred to a 96-well culture plate. Finally, the absorbency at 450 nm was recorded by a microplate reader.

**1.8 RT-PCR**

Gene expression of ECs and SCs on various scaffolds was detected using RT-PCR technique. Total RNA from Schwann cells and endothelial cells was isolated using RNeasy Mini Kit (Qiagen, Valencia, CA, USA) according to the manufacturer’s protocol. The purity and concentration of RNA were determined. Then, 2 mg of superscript III (Life Technologies), total RNA and anchored oligo-dT primers (Operon, Huntsville, AL, USA) were used to perform the reverse transcription. After that, the real-time RTPCR Reaction was performed using an ABI7500 instrument (Applied Biosystems, Foster City, CA, USA). The primer sequences used for Schwann cells were listed as follows: GAPDH sense, 5′-GCAAGTTCAACGGCACAG-3′, GAPDH antisense, 5′- CGCCAGTAGACTCCACGAC -3′, β-actin sense: 5′−CCTCTATGCCAACACAGT-3′, β-actin antisense: 5′-AGCCACCAATCCACACAG-3′, S100 sense:5′-GTTGCCCTCATTGATGTCT-3′, S100 antisense:5′-CTGCTCTTTGATTTCCTCC-3′, Sox10 sense:5′-GAGGAACCTCGCTGCCTGTC-3′, Sox10 antisense: 5′−CCGGGAACTTGTCATCGTCTG-3′, NGF sense: 5′-GCTGGACCCAAGCTCAC-3′, NGF antisense: 5′−CCCTCTGGGACATTGCTATC-3′. The primer sequences used for Endothelal cells were listed as follows: VEGF sense:５ ＇-ＣＧＴＣＣＴＧＴＧＴＧＣＣＣＣＴＡＡＴ-３＇，VEGF antisense: ５＇-ＴＧＧＣＴＴＴＧＧＴＧＡＧＧＴＴＴＧＡＴ-３＇, MMP-2 sense: 5′-GAGTTGGCAGTGCAATACCT-3′, MMP-2 antisense: 5′-CCAAAGAACTTCTGCATCTTCT-3′, Angiogenin sense: 5′-GTCAAGCTTCTGTGTTGGAAGAGATGGTGA-3′, Angiogenin antisense:5′-CCACTCGAGCCGCTGGTTACGGACGACGGA-3′, CD31 sense:5’- GACAGCCAAGGCAGATGCAC -3’,CD31 antisense: 5’- ATTGGATGGCTTGGCCTGAA -3’. GAPDH sense: GTCAGCCGCATCTTCTTTTG, GAPDH antisense: GCGCCCAATACGACCAAATC. The target gene expression was obtained via comparing to the endogenous control GAPDH.

**1.9 Western blot**

Protein expression of both Schwann cells and Endothelial cells was detected using western blot assay. Both cells were firstly cultured on scaffolds for 3 days, then the proteins of the cells were extracted using Radio Immunoprecipitation Assay (RIPA) buffer containing 10mM Tris-HCL, 1mM EDTA, 1% Nonidet P-40, 1% sodium dodecyl sulfate, 50mM sodium fluoride, 1:100 proteinase inhibitor cocktail and 50mM b-glycerophosphate, and measured using a BCA protein assay kit to obtain the total protein level of α-tubulin. After that, the whole cell lysates or subcellular fragments subjected to SDS electrophoresis and further transferred to a polyvinylidene difluoride membrane (0.45 μm, Millipore, Bio-Rad, US). The membranes were then blocked with 5% skim milk for 1 h and incubated with primary antibodies overnight. Subsequently, the membranes were extensively washed with TBST (Tris Buffered Saline Tween-20) three times and incubated with horseradish peroxidase-conjugated secondary antibody (BOSTER, China). Finally, the antigen–antibody complexes were immune-detected using the electrochemiluminescence (ECL) western blotting reagents as recommended by the manufacturer. PDQuest 7.2.0 software (Bio-Rad) software was used to analyze the data.

**1.10 In vivo implantation**

**1.10.1 Animal surgery**

All animals experiment procedures were approved ethically by the Administration Committee of Experimental Animals, Jiangsu Province, China and performed according to the institutional animal care guidelines. Adult SD rats with weight of around 200g were randomly divided into four groups (n=6 for each group): autologous group, defect group, bare conduit group and biofunctionalized conduit group. Firstly, all animals were anesthetized with intraperitoneal injection of sodium pentobarbital (3%, 30 mg/kg body weight), then the sciatic nerve in the left lateral thigh was exposed after skin incision and muscle split. After that, a 10-mm-long defect of sciatic nerve was made by exciting and removing a segment of sciatic nerve. In defect group, the sciatic nerve defect was left without bridging, in bare conduit group and biofunctionalized conduit group, the sciatic nerve defect was bridged using a bare chitosan/collagen (CC) conduit and a VEGF/IKVAV (50:50) immobilized chitosan/collagen (CCIV) conduit, respectively. In autologous group, the cut nerve segment with head and tail upside down was re-implanted into the sciatic nerve defect site. After surgery, all animals were housed and cared routinely, and their general conditions and locomotion activities were monitored daily.

**1.10.2 Vascularization evaluation**

Firstly, the vascularization of controlled CC scaffolds and CCIV scaffolds was evaluated using chicken embryos according to shell-free cultivation protocols in previous study[[2](#_ENREF_2)]. Briefly, the fertilized eggs were incubated at 37℃ for 3 days and opened a window with a diameter of around 1 cm. Then, the pre-sterile scaffolds with the size of 8×5×2 mm were implanted into the egg white with visible capillary network under the chorioallantoic membrane (CAM) and further incubated for 8 days at 37°C in an incubator with humidity of 60% (Nuanlifang, China). After that, the scaffolds were taken out and the developing blood vessels were examined by an optical microscope. The total length of new blood vessels in the scaffolds was measured using image J software and statistically analyzed. Besides, the longitudinal sliced scaffolds with thickness of 10 μm were also stained with CD31 antibody and observed using fluorescence microscope to obtain the morphology of blood vessel. Secondly, the vascularization of the scaffolds was also evaluated after bridging the injured sciatic nerve for 14 days using the following method. Initially, the scaffolds taken from the implantation site were fixed with 4% paraformaldehyde for at least 24h, and then transferred into 30% sucrose solution for 30 min. Then, the scaffolds were immersed into glycerin solution with gradient concentration from 50%, 70%, 85% to 100% in ultrapure water to perform transparency process, 3 h for each concentration. The transparent samples were finally observed under an optical microscope.

**1.10.3 Immunohistochemistry analysis**

At the 14 th day and the 12 th week after surgery, the rats in each group were killed and the tissue or scaffolds at the place of nerve gap was taken and fixed with 4% paraformaldehyde for at least 24 h. Then, the tissue or scaffolds were cut into longitudinal or transverse sections with thickness of 10 μm using a freezing microtome (M1900, Leica, Germany). After that, the sections were sequentially incubated with rabbit anti-NF200 polyclonal antibody (1:150, Sigma-Aldrich) and goat anti-s100β polyclonal antibody (1:150, Sigma-Aldrich) at 4℃ for 24 h, and then further incubated with FITC labeled secondary antibody: goat anti-rabbit IgG (1:200, Gibco) and Tritc labelled rabbit anti goat IgG (1:200, Gibco) at 4 ℃ overnight. Finally, the samples were observed by a fluorescent microscope. In addition, horchest33342 (5μg/mL in PBS) was used to stain the nucleus at room temperature for 30 min. After that, the sections were observed using an inverted fluorescence microscope. The length and the number of the new regenerating nerve fibers in the middle region of a scaffold were determined using Morphometric analysis

**1.10.4 Electrophysiological assessment**

Electrophysiological tests were performed after surgery of 14 days and 12 weeks to evaluate the functional recovery of the injured nerve. In brief, the sciatic nerve at the injured side was firstly re-exposed after anesthesia with sodium phenobarbital. Then, the nerve trunk at the proximal and distal was stimulated with electrical stimuli (10 mV in strength), and the compound muscle action potentials (CMAPs) were measured on the targeted gastrocnemius belly at the ipsilateral side. In addition, the normal CMAP on the contralateral uninjured side was also recorded as control. The motor nerve conduction velocity (MCV) was subsequently calculated in terms of the CMAP amplitude and the distance between two stimulation sites.

**1.10.5 Muscle evaluation**

The muscle wet weight ratio (MWWR) was used to evaluate the muscle recovery adjacent to the repaired sciatic nerve after surgery of 14 days and 12 weeks, respectively. The anterior tibial and gastrocnemius muscles at the injured and contralateral uninjured sides were harvested from deeply anaesthetized animals injured and contralateral uninjured sides, and weighed immediately to determine the wet weight ratio of

Muscles. MWWR=(the wet weight of muscle on the injured side/the wet weight of muscle onthe uninjured side).

**1.10.6 TEM**

TEM was used to observe the myelination formation during nerve regeneration. Briefly, a pre-cooled 2.5% glutaraldehyde was used to fix the sections of regenerated nerves for 3 h, followed by a post-fixation with 1% osmium tetraoxide solution for 1 h. Then, the nerves were washed, dehydrated and embedded in Epon 812 epoxy resin. After that, the nerves were cut into ultra-thin sections with thickness of 60 nm and stained with uranyl acetate and lead citrate. Finally, the sections were observed using a TEM (JEOL Ltd., Tokyo, Japan), ten random fields of each section were captured to statistically determine the diameter of myelinated nerve fibers, the thickness of myelin sheaths and the number of myelin sheath layers using Image Pro Plus software (Media Cybernetics, SilverSpring, MD).

**1.11 Data Analysis.**

All obtained data were expressed as mean ± standard deviation (SD) and analyzed with software SPSS 13.0 software package (Chicago, IL). The statistical evaluation of the data was measured using a Student’s paired t test. The probability (P) value P<0.05 was considered to be statistically significant.

**Reference:**

**[1] G. C. Li, X. Y. Zhao, W. X. Zhao, L. Z. Zhang, C. P. Wang, M. R. Jiang, X. S. Gu, Y. M. Yang. *Biomaterials* 2014,35,8503.**

**[2] V. Djonov, M. Schmid, S. A. Tschanz, P. H. Burri. *Circ Res* 2000,86,286.**

**Figure legends:**

Figure.S1 Sketchmap of the preparation of dual-biofunctionalized chitosan/collagen composite scaffolds.

Figure.S2 Characterization of scaffolds preparation. (A) Ultraviolet (UV) spectra of collagen (CO), chitosan (CS) and chitosan/collagen (CC), (B) Toluidine blue (TBO) staining, (C) Distribution of heparin, and (D) Heparin amount, *P<0.05 versus other samples.

Figure.S3 Immobilization amount and release behavior of vascular endothelial growth factor (VEGF) (A-C) and Ile-Lys-Val-Ala-Val (IKVAV) (D-F) in various scaffolds. *P<0.05, **P<0.05 versus other samples.


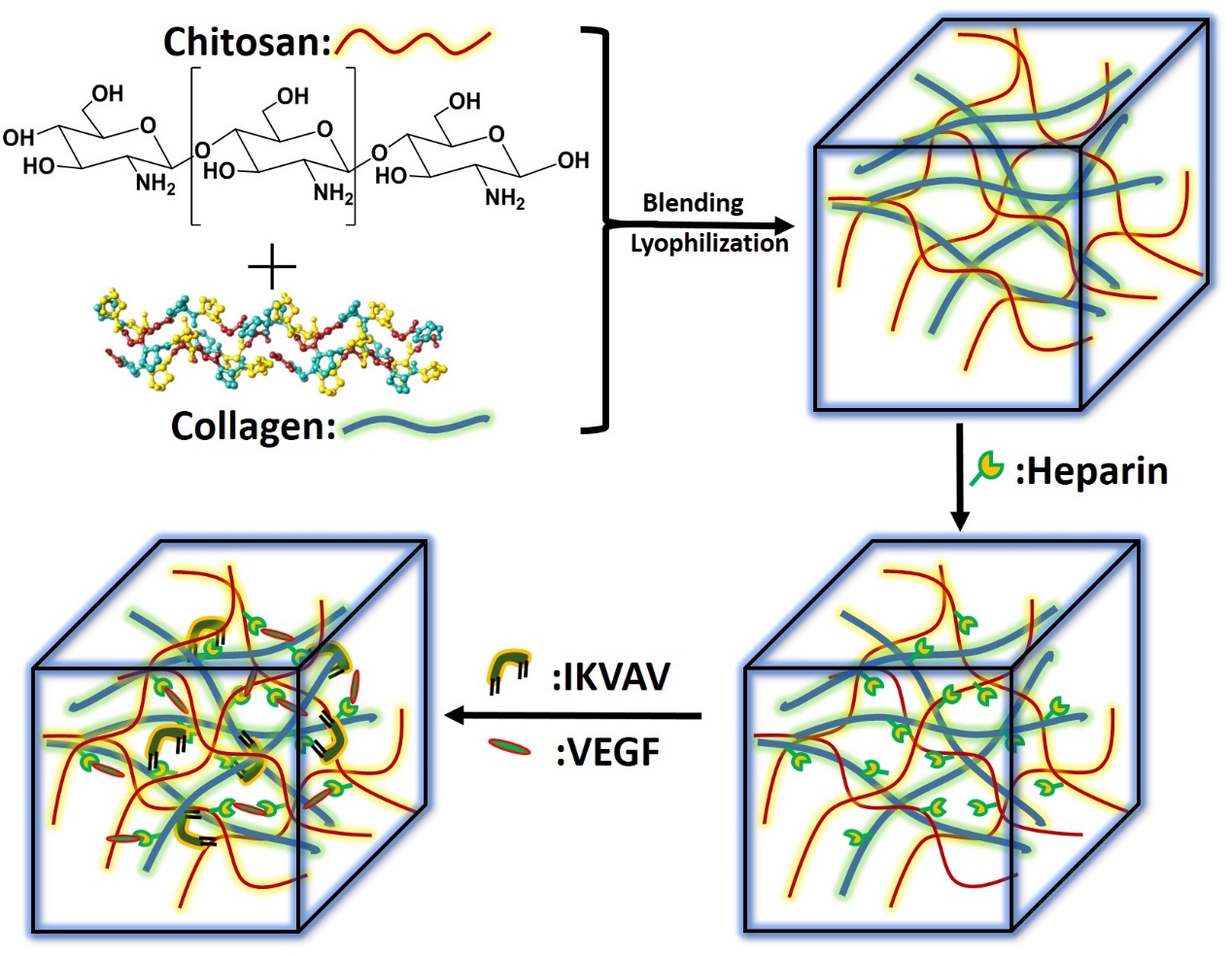


Figure.S1


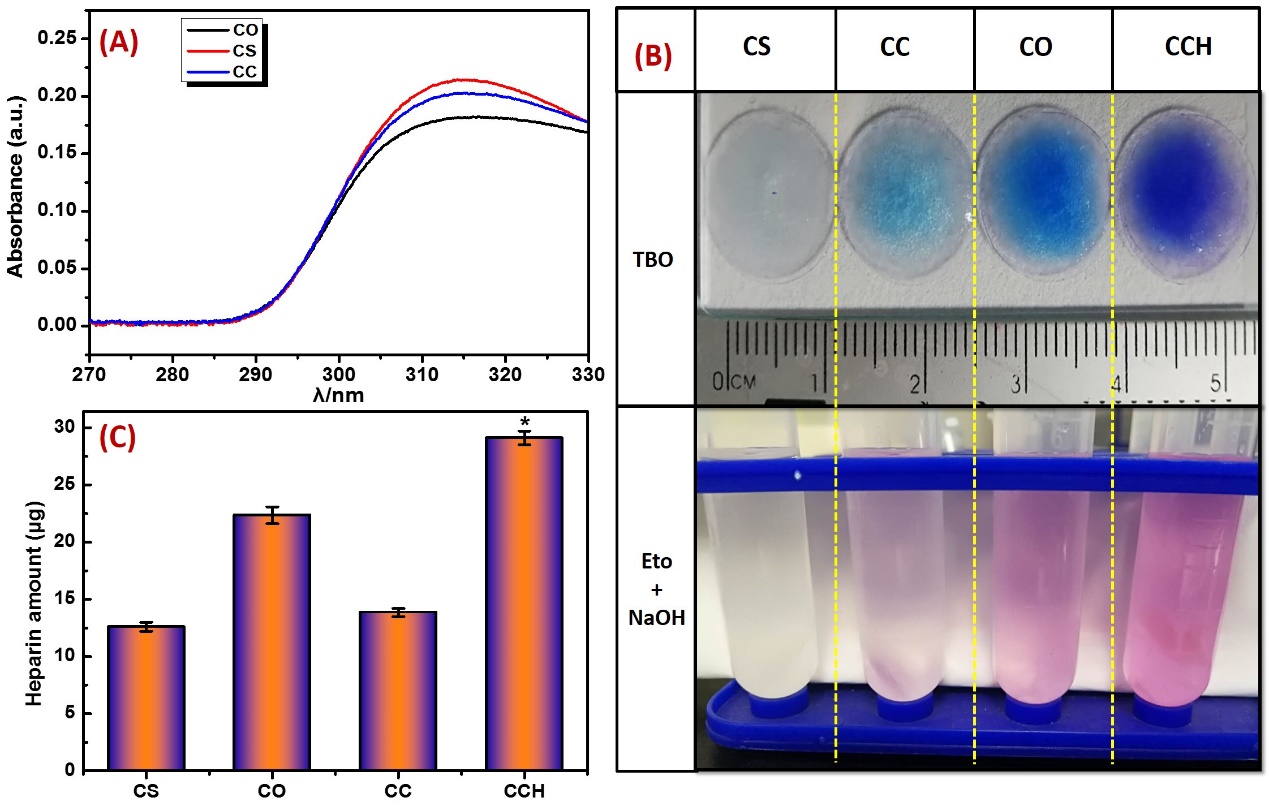


Figure.S2


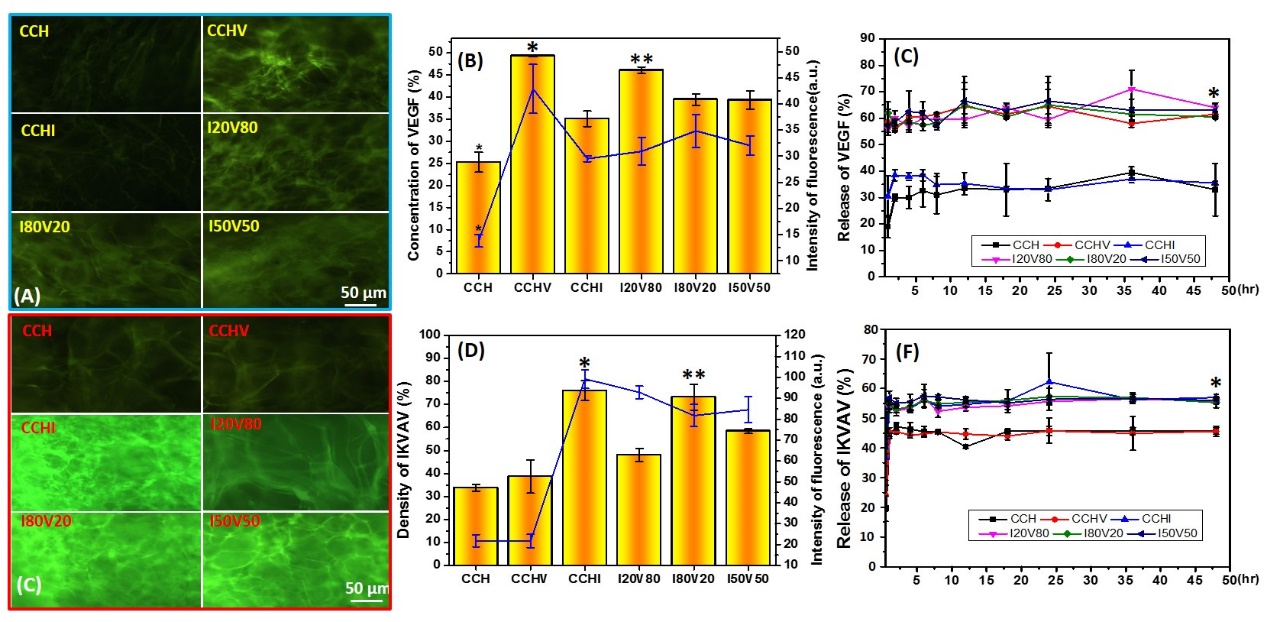


Figure.S3
